# Normal Values for Segmental Bioimpedance Spectroscopy in Pediatric Patients

**DOI:** 10.1371/journal.pone.0126268

**Published:** 2015-04-13

**Authors:** Maria Laura Avila, Leigh C. Ward, Brian M. Feldman, Madeline I. Montoya, Jennifer Stinson, Alex Kiss, Leonardo R. Brandão

**Affiliations:** 1 Department of Paediatrics, The Hospital for Sick Children, University of Toronto, Toronto, Ontario, Canada; 2 School of Chemistry and Molecular Biosciences, The University of Queensland, St. Lucia, Brisbane, Australia; 3 Institute of Health Policy, Management and Evaluation, University of Toronto, Toronto, ON, Canada; 4 Child Health Evaluative Sciences, The Hospital for Sick Children, University of Toronto, Toronto, Ontario, Canada; 5 Lawrence S. Bloomberg Faculty of Nursing, University of Toronto, Toronto, Ontario, Canada; 6 Department of Research Design and Biostatistics, Sunnybrook Health Sciences Centre, Toronto, ON, Canada; Medical University of Graz, AUSTRIA

## Abstract

**Introduction:**

Localized limb edema is a clinically relevant sign in diseases such as post-thrombotic syndrome and lymphedema. Quantitative evaluation of localized edema in children is mainly done by measuring the absolute difference in limb circumference, which includes fat and fat-free mass. Bioimpedance spectroscopy (BIS) provides information on the fluid volume of a body segment. Our objective was to determine normal ranges for segmental (arm and leg) BIS measurements in healthy children. Additionally, we determined the normal ranges for the difference in arm and ankle circumference and explored the influence of handedness and the correlation between techniques.

**Methods:**

Healthy children aged 1-18 years were recruited. The ratio of extracellular fluid content between contralateral limbs (estimated as the inter-arm and inter-leg extracellular impedance ratio), and the ratio of extracellular to intracellular fluid content for each limb (estimated as the intracellular to extracellular impedance ratio) were determined with a bioimpedance spectrometer. Arm and ankle circumference was determined with a Gulick II tape.

**Results:**

We recruited 223 healthy children (48 infants, 54 preschoolers, 66 school-aged children, and 55 teenagers). Normal values for arm and leg BIS measurements, and for the difference in arm and ankle circumference were estimated for each age category. No influence of handedness was found. We found a statistically significant correlation between extracellular impedance ratio and circumference difference for arms among teenagers.

**Conclusion:**

We determined normal BIS ranges for arms and legs and for the difference in circumference between arms and between ankles in children. There was no statistically significant correlation between extracellular impedance ratio and difference in circumference, except in the case of arms in adolescents. This may indicate that limb circumference measures quantities other than fluid, challenging the adequacy of this technique to determine the presence of localized edema in most age groups.

## Introduction

Post-thrombotic syndrome (PTS) is a potential long-term complication of deep vein thrombosis (DVT) estimated to affect 1 in 3 adults [1] and 1 in 4 children [2] who have sustained limb DVT.

Since localized edema is a typical clinical feature of PTS, all clinical tools and criteria used to assess PTS in both adult and pediatric patients include the measurement of edema [3–9].

Detection of localized edema is important for the diagnosis and follow-up not only of PTS, but also of other conditions that feature excessive limb fluid content, such as other forms of chronic venous disease and lymphedema[10]. Moreover, early diagnosis of segmental edema has been shown to allow timely therapeutic interventions and better results in patients with lymphedema[11].

Currently, the assessment tools most commonly used in the clinical setting for objective evaluation of localized edema are based on the measurement of either *limb circumference* or *limb volume* [12]. The measurement of *limb circumference* involves determining the difference between the circumferences of both limbs at one or two anatomical points, using a measuring tape. Limb circumference determination has been used predominantly to assess edema in children at risk of PTS [3, 13–15]. *Limb volume* can be assessed directly or indirectly using a number of techniques, the four most common being the use of a measuring tape and mathematical formulae, water displacement [10, 16], perometry, and bioimpedance. Although these methods have been shown to be accurate and precise for the assessment of limb volume in adult patients affected by lymphedema [17], less is known about their accuracy in children.

Each method has advantages and disadvantages. Limb volume determination with measuring tape is inexpensive and highly acceptable to patients and clinicians, but it can be tedious and time-consuming to perform, and identification of bony landmarks can be difficult in some patients [18]. Water displacement is a low cost technique, but can be time-consuming, sensitive to movement artefacts, and may be associated with infection control problems [19, 20]. Perometry is a rapid, accurate, and precise method, but the equipment is very expensive, and may not be suitable for young patients since they have to adopt a stable position for the measurement[17].

Importantly, the major limitation of these three methods is that they provide a measurement of *total limb volume*, and an increase in volume is then inferred to reflect limb edema. However, the total volume of a limb is composed of not only fluid content, but also fat and muscle. Bioimpedance spectroscopy (BIS) is able to provide information on the amount of extracellular, intracellular, and total fluid content of a body segment, therefore avoiding the inaccuracy introduced by changes in fat and muscle tissue in a swollen limb [17]. An additional advantage of BIS is that several determinations can be taken in a few seconds, which is particularly important in pediatric patients, who may find it difficult to stay still.

The main objective of our study was to determine the reference ranges for BIS measurements for upper and lower limbs (i.e., arms and legs) in a population of healthy children. The secondary outcomes comprised 1) determination of reference ranges for the difference in circumference between both arms and both ankles in these patients, 2) investigation of the influence of handedness on BIS measurements, and on the difference in arm and ankle circumference, and 3) determination of the correlation between BIS measurements and the difference in circumference.

## Methods

Healthy children aged 1–18 years were recruited in this cross-sectional study that was conducted at the Hospital for Sick Children (pilot), and at the Ontario Science Centre, Toronto, ON.

Four age-categories were included: toddlers (1–2 year-olds), pre-schoolers (3–4 year-olds), school-aged children (5–11 year-olds), and adolescents (12–18 year-olds).

Ethics statement: The University of Toronto and the Hospital for Sick Children Research Ethics Boards approved the study. Written informed consent was obtained from parents/legal guardians or participants, as appropriate.

The following variables were collected: age, sex, height, weight, handedness, BIS measurements, and anatomical measurements (arm and ankle circumference, as described below).

1. Height was measured using a portable stadiometer to the nearest 0.1 cm; weight was measured with a digital scale to the nearest 0.1 kg. Handedness was determined as per parental or participant’s report. Body mass index percentile or weight-for-length percentile was estimated in participants older or younger than 2 years, accordingly. Body mass index percentiles were estimated using a SAS macro available through the Centers for Disease Control and Prevention website [21].
2. Biompedance spectroscopy measurements: the principle underlying bioimpedance is that the opposition of biological tissues to the flow of imperceptible electrical current passing through is inversely proportional to the fluid volume of the tissue [22]. Biompedance spectroscopy involves the application of a range of frequencies (usually from around 3 kHz to 1 MHz) and the measurement of the impedance or resistance to the passage of this electric current. Whereas the impedance at low frequencies, ideally at zero frequency (R0), reflects the extracellular fluid content alone, the impedance at high frequencies, ideally at infinite frequency (Rinf), reflects total fluid content. Both R0 and Rinf are used to derive Ri, the impedance of the intracellular fluid compartment. Segmental impedance values are determined from impedance measurements made of the body segments according to the principle of equipotentials [23]. Importantly, the measurement of extracellular fluid content of an arm or leg (reflected by the R0 value) is normalized by comparison to the value of the contralateral limb, and expressed as a ratio [24]. The normalization of the R0 values allows accounting for the wide individual biological variation in impedance values, which depends on factors such as length of the arms or legs, exercise, and diet [25]. Hence, it follows that the R0/R0 ratio is only useful in patients with unilateral arm or leg edema [26–28]. However, when patients have bilateral arm or leg edema, the extracellular fluid content can be normalized against the intracellular fluid of the same limb, and because resistance is inversely related to volume, the extracellular to intracellular fluid volume is calculated as the inverse ratio of their resistances (extracellular/intracellular fluid content = intracellular/extracellular impedance ratio = Ri/R0 ratio) [24, 25].
  Segmental BIS (arm: wrist to axilla; leg: ankle to groin) was measured following standard protocols, in accordance with the principle of equipotentials [23, 29]. At the time of the testing, the subjects were asked to remove all jewellery (except rings and earrings) and shoes; skin was prepared with an alcohol wipe and allowed to dry prior to placing the surface electrode. Participants aged 3 years and older were positioned lying supine on a non-conductive examination table, arms at sides with palms facing down, and legs slightly abducted. Toddlers were allowed to sit up during measurements to encourage cooperation. Lightly adhesive silver/silver chloride EKG-style resting electrodes were placed as follows: 1) voltage sensing electrodes were placed on the dorsum of the wrists adjacent to the ulnar styloid process, and anterior to the ankle joints between the malleoli; 2) current drive electrodes were placed distal to the voltage sensing electrodes, on the dorsum of the hands and feet. Impedance measurements were performed with an SFB7 bioimpedance spectrometer (ImpediMed Ltd, Brisbane, Australia). Device calibration was checked regularly, following the manufacturer’s instructions.
3. Circumference measurements were determined with a Gulick II anthropometric tape, a no-stretch retractable tape with a tensioning device that allows application of constant tension, thus minimizing measurement error that can be ascribed to differences in the applied tension[30].
  - Arm circumference was measured at a point equidistant to the acromium process and the olecranon process. This technique was chosen based on a previous pilot study conducted at our institution, which showed better intra and inter-rater reliability using this technique (unpublished data). Measurements were recorded in cm, rounded to the nearest first decimal place. The absolute difference in cm between right and left arm and between dominant and non-dominant arm were estimated.
  - Given the exposed setting of the study, and for privacy reasons, we decided to measure the ankles of the participants following the “Figure-of-8” technique [30] instead of measuring mid-calf and mid-thigh circumferences. For this measurement, the participant was positioned lying supine on a table, and (whenever possible) keeping the ankle in neutral dorsiflexion. The Gulick II anthropometric tape was placed midway between the tendon of the tibialis anterior and the lateral malleolus, pulled medially toward the tuberosity of the navicular, then pulled laterally across the longitudinal arches of the foot toward the base of the 5th metatarsal. The tape was then pulled around the ankle joint, encircling an imaginary line below the medial malleolus, across the Achilles tendon and below the lateral malleolus, ending where the measurement started. A video showing how this technique is performed is available [31]. The results were recorded in cm, rounded to the nearest first decimal place. The absolute difference in cm between right and left ankle and between dominant and non-dominant ankle were estimated.

It is of note that arm and ankle circumference measurements of the healthy subjects who were recruited at the Hospital for Sick Children to participate in the study pilot were not taken.

A trained researcher performed all the anatomical measurements and a second trained researcher undertook all BIS determinations. Both researchers were blinded to each other’s results.

### Data Analysis

Categorical data were summarized as percentages and ratios. Means and standard deviations (SD) or medians and interquartile ranges (IQR) were used to report continuous data depending on data distribution.

The BIS data were processed using the manufacturer’s software (Bioimp version 5.4.0.3, Copyright© 2012 ImpediMed) to obtain extracellular (R0) and intracellular (Ri) resistance values for each limb. The R0/R0 and Ri/R0 ratios for arms and legs were estimated from these values.

The coefficient of variation for replicate R0 and Ri measurements was estimated. The percentile distributions of the R0/R0 ratios for arms and legs and of the difference in circumference for arms and ankles were determined in relation to age group.

The impact of handedness on R0/R0 ratios was investigated within each group category by comparing the right limb R0 to left limb R0 ratio vs. the dominant hand side R0 to non-dominant hand side R0 ratio using paired t-tests. A similar analysis was performed to compare the impact of dominance on the difference in circumferences (right—left circumference and dominant—non-dominant hand side). The potential effect of sex on R0/R0 ratios was explored using t-tests.

The effect of age and hand side dominance on Ri/R0 ratios of each limb was analyzed with two-way analysis of variance (ANOVA).

Lastly, the relationship between arm R0/R0 ratios and arm circumference difference, and between leg R0/R0 ratios and ankle circumference difference was estimated in each age group using the Pearson product-moment correlation coefficient.

Statistical significance was set at p = 0.05.

SAS software, version 9.2 of the SAS System for Windows, copyright© 2008, SAS Institute Inc., Cary, NC, USA and MedCalc Statistical Software, version 13.1.2, were used for data analyses.

## Results

Two hundred and twenty three healthy children were recruited during the study period. Two percent of the population (n = 6) was recruited at the Hospital for Sick Children, and the remaining participants (n = 217) were recruited at the Ontario Science Centre, Toronto, Ontario. Age distribution was as follows: 48 infants, 54 preschoolers, 66 school-aged children, and 55 teenagers. The general characteristics of the cohort are presented in Table 1.

**Table 1:** General Characteristics Of The Cohort.

**Male:female ratio**	Toddlers	1:1
	Preschoolers	1.1:1
	School age	1.3:1
	Teenagers	0.8:1
**Weight-for-length percentile for <2 year-olds, median (IQR)**	Toddlers n = 20	64 (76)
**BMI percentile for ≥2 year-olds, median (IQR)**	Toddlers n = 28	62 (33)
	Preschoolers	53 (50)
	School age	44 (47)
	Teenagers	61 (52)
**Handedness, right/left (% left handed)**	Preschoolers	46/4 (8)
	School age	53/13 (27)
	Teenagers	48/7 (13)

Legend: IQR refers to interquartile range; BMI, to body mass index.

The coefficient of variation of R0 ranged between 0.33% and 0.46%, whereas that of Ri ranged between 1.46% and 4.00%. The distribution of R0/R0 ratios for arms and legs per age category is displayed in Table 2.

**Table 2:** Extracellular Resistance (R0/R0) Ratios For Arms And Legs By Age Group.

Age group	n	Mean	SD	P3	P10	P25	P50	P75	P90	P97	Segment
**Toddlers**	48	1.01	0.047	0.93	0.96	0.98	1.00	1.03	1.08	1.12	**Right/Left Arm**
**Preschoolers**	54	1.01	0.037	0.94	0.95	0.98	1.00	1.03	1.06	1.07	
**School age**	66	1.01	0.033	0.95	0.96	0.98	1.01	1.03	1.04	1.08	
**Teenagers**	55	0.99	0.044	0.92	0.94	0.95	0.98	1.01	1.04	1.09	
**Toddlers**	48	1.01	0.048	0.93	0.96	0.98	1.00	1.03	1.08	1.12	**Dominant/Non-dominant Arm**
**Preschoolers**	54	1.01	0.036	0.94	0.96	0.99	1.01	1.03	1.03	1.07	
**School age**	66	1.01	0.033	0.95	0.97	0.98	1.01	1.03	1.04	1.08	
**Teenagers**	55	0.99	0.045	0.92	0.94	0.96	0.99	1.02	1.05	1.09	
**Toddlers**	48	1.00	0.053	0.91	0.94	0.97	0.99	1.03	1.09	1.11	**Right/Left Leg**
**Preschoolers**	54	1.00	0.034	0.93	0.96	0.98	1.00	1.01	1.03	1.07	
**School age**	66	0.99	0.025	0.95	0.96	0.97	0.99	1.00	1.02	1.05	
**Teenagers**	55	1.00	0.032	0.95	0.96	0.97	0.99	1.02	1.03	1.07	
**Toddlers**	48	1.00	0.053	0.91	0.95	0.97	0.99	1.04	1.09	1.11	**Dominant/Non-dominant Leg**
**Preschoolers**	54	1.00	0.034	0.93	0.96	0.98	1.00	1.01	1.03	1.07	
**School age**	66	0.99	0.026	0.95	0.96	0.97	0.99	1.01	1.02	1.04	
**Teenagers**	55	0.99	0.031	0.93	0.95	0.97	0.99	1.02	1.03	1.05	

Legend: SD refers to standard deviation, P3 to the 3^rd^ percentile, P10 to the 10^th^ percentile, P25 to the 25^th^ percentile, P50 to the 50^th^ percentile, P75 to the 75^th^ percentile, P90 to the 90^th^ percentile, and P97 to the 97^th^ percentile.

Regarding the influence of handedness on R0/R0 ratios, we found little variation in the distribution of values when comparing the right/left limb vs. dominant/non-dominant hand side. Indeed, paired t-tests showed no statistically significant difference between these two methods to estimate arm and leg R0/R0 ratios (toddlers t = 1.0, 47 degrees of freedom [df], p = 0.32; preschoolers t = -1.8, 53 df, p = 0.08; school aged children t = -0.9, 65 df, p = 0.38; teenagers t = -1.0, 54 df, p = 0.30 for arms; toddlers t = 1.0, 47 df, p = 0.32; preschoolers t = -1.2, 53 df, p = 0.23; school aged children t = -0.8, 65 df, p = 0.43; teenagers t = 0.42, 54 df, p = 0.68 for legs).

Of note, we found no statistically significant difference in arm or leg R0/R0 ratios between males and females (arms t = 1.41, 221 df. p = 0.14; legs t = 0.32, 218 df, p = 0.75).

Table 3 shows the distribution of extracellular to intracellular fluid content, expressed as Ri/R0 ratio, for each body segment in each age category. There was a statistically significant difference in the Ri/R0 ratios among age groups, with no significant effect of handedness on any of the limbs (Table 3).

**Table 3:** Extracellular To Intracellular Resistance (Ri/R0) Ratios By Body Segment.

Age group	n	Mean	SD	P3	P10	P25	P50	P75	P90	P97	Segment	Two-Way ANOVA*
**Toddlers**	48	3.33	0.50	2.17	2.67	3.06	3.39	3.66	3.85	4.21	**Right Arm**	Age group: F _3,217_ = 32.7, MSE = 5.1, p <0.001; Handedness: F _2,217_ = 2.7, MSE = 0.4, p = 0.07
**Preschoolers**	54	3.26	0.35	2.47	2.79	3.06	3.31	3.52	3.41	3.89		
**School age**	66	2.94	0.37	2.17	1.49	2.71	2.88	3.12	3.38	4.03		
**Teenagers**	55	2.66	0.38	1.98	2.17	2.38	2.66	2.99	3.16	3.34		
**Toddlers**	48	3.43	0.48	2.68	2.79	3.05	3.44	3.79	3.96	4.27	**Left Arm**	Age group: F _3,217_ = 27.0, MSE = 4.4, p <0.001; Handedness: F _2,217_ = 1.88, MSE = 0.31, p = 0.15
**Preschoolers**	54	3.39	0.38	2.59	2.93	3.15	3.37	3.63	3.78	4.06		
**School age**	66	3.05	0.35	2.39	2.60	2.83	3.03	3.21	3.48	3.88		
**Teenagers**	55	2.82	0.43	2.05	2.31	2.54	2.76	3.04	3.43	3.72		
**Toddlers**	48	3.93	0.66	3.00	3.19	3.56	3.83	4.27	4.59	5.13	**Right Leg**	Age group: F _3,217_ = 56.8, MSE = 16.6, p <0.001; Handedness: F _2,217_ = 0.24, MSE = 0.07, p = 0.79
**Preschoolers**	54	3.35	0.45	2.46	2.75	3.16	3.38	3.61	3.89	4.27		
**School age**	66	2.84	0.52	1.91	2.40	2.69	2.89	3.14	3.37	3.76		
**Teenagers**	55	2.67	0.54	1.95	2.13	2.36	2.73	3.01	3.30	3.67		
**Toddlers**	48	4.11	0.80	2.44	3.28	3.60	4.18	4.65	4.95	5.33	**Left Leg**	Age group: F _3,217_ = 39.7, MSE = 12.3, p <0.001; Handedness: F _2,217_ = 0.16, MSE = 0.08, p = 0.82
**Preschoolers**	54	3.63	0.63	2.57	2.95	3.25	3.56	3.85	4.25	5.40		
**School age**	66	3.06	0.54	1.98	2.52	2.81	3.12	3.32	3.60	4.13		
**Teenagers**	55	2.87	0.63	2.08	2.21	2.47	2.89	3.28	3.18	4.13		

Legend: SD refers to standard deviation, MSE, to mean square error, P3 to the 3^rd^ percentile, P10 to the 10^th^ percentile, P25 to the 25^th^ percentile, P50 to the 50^th^ percentile, P75 to the 75^th^ percentile, P90 to the 90^th^ percentile, and P97 to the 97^th^ percentile

*Two-way ANOVA analyzing the effect of two factors, age group and hand side dominance on Ri:R0 ratios.

The distribution of the difference in circumference for arms and for ankles is shown in Table 4. As observed with R0/R0 ratios, there was little impact of hand side dominance on the distribution of values of circumference difference: paired t-tests showed no statistically significant difference when comparing right minus left limb vs. dominant minus non-dominant hand side (toddlers t = -1.2, 47 df, p = 0.25; preschoolers t = -0.7, 53 df, p = 0.51; school aged children t = -1.4, 64 df, p = 0.18; teenagers t = 0.7, 50 df, p = 0.45 for arms; toddlers t = -1.0, 47 df, p = 0.32; preschoolers t = -1.7, 53 df, p = 0.10; school aged children t = 1.25, 64 df, p = 0.22; teenagers t = 1.16, 50 df, p = 0.25 for legs).

**Table 4:** Difference In Circumference (In cm) For Arms And Ankles.

Age group	n	Mean	SD	P3	P10	P25	P50	P75	P90	P97	Segment
**Toddlers**	48	-0.08	0.48	-1.00	-0.70	-0.45	-0.05	0.20	0.50	1.00	**Right—Left Arm**
**Preschoolers**	54	-0.08	0.52	-1.00	-0.70	-0.40	-0.10	0.40	0.60	1.00	
**School age**	64	-0.08	0.46	-0.90	-0.70	-0.30	0.00	0.20	0.50	1.00	
**Teenagers**	51	0.03	0.77	-1.40	-0.80	-0.50	0.00	0.60	1.00	1.60	
**Toddlers**	48	-0.08	0.48	-1.00	-0.70	-0.45	-0.05	0.20	0.50	1.00	**Dominant—Non-dominant Arm**
**Preschoolers**	54	-0.07	0.52	-1.00	-0.70	-0.40	-0.10	0.30	0.60	1.00	
**School age**	64	0.05	0.47	-0.90	-0.50	-0.20	0.00	0.30	0.50	1.30	
**Teenagers**	51	0.10	0.77	-1.10	-0.70	-0.50	0.00	0.60	1.10	1.60	
**Toddlers**	48	-0.11	0.70	-1.80	-1.00	-0.50	0.00	0.25	0.70	1.00	**Right—Left Ankle**
**Preschoolers**	54	-0.06	0.45	-1.00	-0.50	-0.30	0.00	0.20	0.50	1.00	
**School age**	64	0.06	0.48	-1.00	-0.50	-0.20	0.00	0.40	0.60	1.00	
**Teenagers**	51	-0.01	0.67	-1.00	-0.80	-0.50	0.00	0.50	0.65	1.50	
**Toddlers**	48	-0.09	0.70	-1.80	-1.00	-0.50	0.00	0.40	0.70	1.00	**Dominant—Non-dominant Ankle**
**Preschoolers**	54	-0.01	0.45	-1.00	-0.50	-0.20	0.00	0.20	0.50	1.00	
**School age**	64	0.00	0.48	-1.00	-0.50	-0.35	0.00	0.25	0.50	1.00	
**Teenagers**	51	-0.09	0.66	-1.50	-1.00	-0.50	0.00	0.40	0.50	1.40	

Legend: SD refers to standard deviation, P3 to the 3^rd^ percentile, P10 to the 10^th^ percentile, P25 to the 25^th^ percentile, P50 to the 50^th^ percentile, P75 to the 75^th^ percentile, P90 to the 90^th^ percentile, and P97 to the 97^th^ percentile.

The correlation between R0/R0 ratios and circumference difference was statistically significant in the case of arm measurements in teenagers (r = -0.44; 95% confidence interval [CI] -0.64 to -0.18; p = 0.001). It must be pointed out that the negative direction of the correlation is due to the fact that resistance is inversely related to volume, as explained in the Methods section. Conversely, correlations between arm R0/R0 ratio and difference in circumference in the remaining age groups were not statistically significant (toddlers r = -0.26; 95% CI -0.51 to 0.02; p = 0.07; preschoolers r = -0.15; 95% CI -0.40 to 0.12; p = 0.28, school aged children r = 0.01; 95% CI -0.23 to 0.26; p = 0.92). Similarly, we found no significant correlation between leg R0/R0 ratios and difference in ankle measurement in any age group (toddlers r = 0.15; 95% CI -0.14 to 0.42; p = 0.30; preschoolers r = -0.08; 95% CI -0.34 to 0.19; p = 0.57, school aged children r = 0.00; 95% CI -0.24 to 0.25; p = 0.99, teenagers r = 0.06; 95% CI -0.22 to 0.33; p = 0.67).

## Discussion

We determined the normal R0/R0 and Ri/R0 ratios for arms and legs in pediatric patients, as well as the normal difference in circumference between arms and between ankles.

The R0/R0 ratios for arms that we observed in the pediatric population were similar to those reported in adult patients by Ward et al [27]. In both studies, the mean R0/R0 ratios for arms were slightly closer to one (unity) than those found by Ridner et al [28] and Cornish et al [32] in smaller study samples, perhaps reflecting that the effect of limb dominance has yet to assert itself in the young. Leg BIS R0/R0 ratios in the present cohort were also similar to those reported in healthy adults, especially in the group of teenagers, whose values were slightly below one [26].

Our findings showed that younger patients had a greater extracellular fluid content, as reflected by higher Ri/R0 ratios. This is in keeping with the physiological differences in extracellular and intracellular compartments described in pediatric patients [33]. In addition, the body water content of the fat-free mass has been reported to decline throughout childhood, reaching adult values around the age of 20 years [34]. This concept is consistent with the decline in ratios we found over the four age groups. Interestingly, adult patients have also been described to show extracellular fluid compartment expansion (i.e., higher Ri/R0 ratios) with age [25]. According to the literature, the difference in Ri/R0 ratios in adults is more marked in the legs, suggesting that, unlike the pediatric population, the increase in the amount of extracellular fluid in the elderly may be associated with the age-related increase in the frequency of vascular disorders [25].

Measurement of *limb circumference* has been the standard method to determine the presence of edema not only in pediatric PTS, but also in other diseases characterized by the presence of localized fluid accumulation, such as lymphedema. As regards the difference in circumference, Goldenberg et al [13, 14] described a cohort of 78 healthy children aged 1 to 21 years divided in three age categories (1–5 year-olds n = 30; 6–12 year-olds n = 28; 13–21 year-olds n = 20), and observed that the normal upper limit for the absolute difference between right and left arm circumference (measured at mid-upper arm and mid-forearm) and between right and left leg circumference (measured at mid-thigh and mid-calf) was one cm, irrespective of the age group. Cut-off points were determined as [median + (1.5 x interquartile range)]. In our study, the 97^th^ percentile for difference in arm and ankle measurements in children aged 12–18 years was slightly higher (1.6 cm for arms and 1.4 cm for ankles). The higher upper limits for difference in circumference in our study were also observed when estimating the cut-off point in keeping with Goldenberg et al. The difference could be partly explained by the larger sample size in our study.

We observed no correlation between arm circumference difference and arm R0/R0 measurements, or between ankle circumference difference and leg R0/R0 ratio in 1 to 11 year-olds. A possible explanation to this finding might be that these methods do not measure the same elements of body composition [22]. Whereas R0/R0 ratios reflect extracellular fluid content, the difference in circumference includes all tissues (i.e., fat and fat-free mass). Therefore, a difference detected in the total circumference between arms or ankles is not necessarily due to a difference in fluid content, suggesting that reliance on circumference measurements in the assessment of segmental edema may be misleading in most age groups.

Of note, there is a well-recognised and consistently reported bilateral asymmetry (*directional asymmetry*) in the human body that favours the right side of right-handers and is more pronounced in the arms [35–37]. Directional asymmetry is tied to limb preference and is attributed to differential biomechanical stress during bone growth, due to muscular development [35, 38–43]. As a result, it is not surprising that directional asymmetry has been reported to gradually develop during childhood, and to be more marked in adults than in children [36, 37, 44]. Indeed, a greater right-sided asymmetry in triceps skinfold [38], bone-free lean tissue, bone mineral density [45], and in limb bone dimensions [35] has been reported in adolescent and adults, particularly among males. The effect of sex hormone stimulation during puberty drives lipogenesis, muscle tissue hypertrophy, and bone growth [46], thus accentuating the arm asymmetry among teenagers. Importantly, changes in the relative amounts of adipose and lean tissue may affect the extracellular and intracellular fluid volumes as measured by BIS, since increases in adipose tissue can contribute to expansion of the extracellular water compartment; this could, in part, explain the significant correlation between arm circumference difference and extracellular (R0) resistance ratio among adolescents shown by our results.

The present study showed no conclusive influence of hand side dominance on any of the measurements, which could be explained by the small number of left-handed participants in the study population, the less pronounced directional asymmetry in children in general, as well as the less pronounced effect of directional asymmetry that has been described in left-handed, as opposed to right-handed children and adults [36].

Our results need to be interpreted considering possible limitations to our methods. Though foot dominance was not recorded, it is known that there is a high correlation between hand and foot dominance, and only 5% of people show combined or cross-lateral dominance [47, 48]. Secondly, the population studied here is not representative of obese patients, who have been reported to have a larger extracellular/intracellular water ratio [49]. Hence, further studies focusing on the impact of obesity on Ri/R0 ratios are warranted. Thirdly, unlike the older children, infants would often only cooperate if they were allowed to sit up during BIS measurements and the protocol was modified accordingly. It must be pointed out however, that even though it is not known whether this position has an impact on BIS results, it is unlikely that it significantly affects hydrostatic pressure, and any effect should be similar in both extremities, therefore not affecting the inter-limb ratios. Lastly, whereas we did not assess biological maturity using specific methods, we grouped the children according to calendar age in an attempt to control this factor, which could potentially influence the measured variables.

To conclude, we determined normal segmental BIS ranges for arms and legs, and the normal ranges for the difference in circumference between arms and ankles in pediatric patients. We only found a significant correlation between arm R0/R0 ratios and difference in arm circumference in teenagers, suggesting that BIS and measuring tape measure different elements of body composition.
